# Vertebral artery stump syndrome: A 7-year follow-up case report^☆^

**DOI:** 10.1016/j.radcr.2022.05.063

**Published:** 2022-06-17

**Authors:** Michiru Katayama, Hideki Endo, Megumi Matsuda, Kenji Kamiyama, Toshiaki Osato, Hirohiko Nakamura

**Affiliations:** Department of Neurosurgery, Nakamura Memorial Hospital, South 1, West 14, Chuo-ku, Sapporo, Hokkaido 060-8570, Japan

**Keywords:** Acute ischemic stroke, Angiography, Follow-up, Posterior circulation, Vertebral artery, Vertebral artery stump syndrome

## Abstract

Vertebral artery stump syndrome is rare, but one of the most important causes of posterior circulation stroke. To our knowledge, no optimal treatment for vertebral artery stump syndrome has been established, and there are no reports of long-term follow-up. We describe a 69-year-old man with vertebral artery stump syndrome who attended our hospital because of vertigo. Magnetic resonance imaging detected right cerebellar infarcts. Digital subtraction angiography revealed severe stenosis (functional obstruction) at the origin of the right vertebral artery, with distal antegrade collateral flow from the deep cervical artery. We started him on argatroban and cilostazol, but symptoms recurred after 1 month. We changed from cilostazol to aspirin and clopidgrel, then terminated aspirin 1 month after recurrence. He continued on clopidgrel, and follow-up after 7 years showed no recurrence, including asymptomatic lesions.

## Introduction

Vertebral artery stump syndrome (VASS) is a rare condition but one of the most important causes of posterior circulation stroke [1,2]. VASS is also associated with a high risk of recurrent stroke and is potentially fatal. The optimal treatment for VASS has not yet been established and, to the best of our knowledge, there are no reports of long-term follow-up. Herein, we report a rare case of VASS with a 7-year follow-up period.

## Case report

**Fig. 1:**
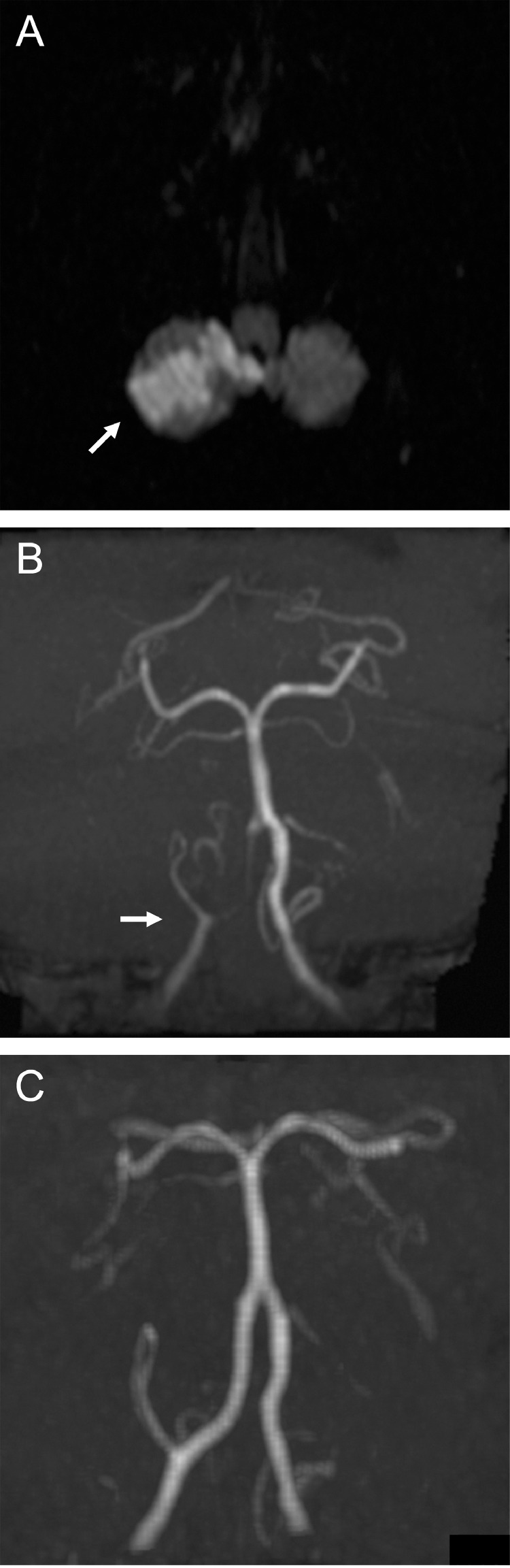
Magnetic resonance imaging showing right cerebellar infarcts (arrow) (A). Magnetic resonance angiography demonstrating impaired delineation of the right vertebral artery (arrow) (B). Adequate delineation had been obtained 1 year previously (C).

A 69-year-old man with a history of hypertension, dyslipidemia, and gout visited our hospital because of vertigo. At the time of visiting, his symptoms had improved and no ataxia was noted. Magnetic resonance imaging (MRI) detected right cerebellar infarcts (Fig. 1A). Magnetic resonance angiography (MRA) showed impaired delineation of the right vertebral artery (VA), even though adequate delineation had been obtained 1 year previously (Fig. 1B, C). He was urgently admitted to our hospital.

**Fig. 2:**
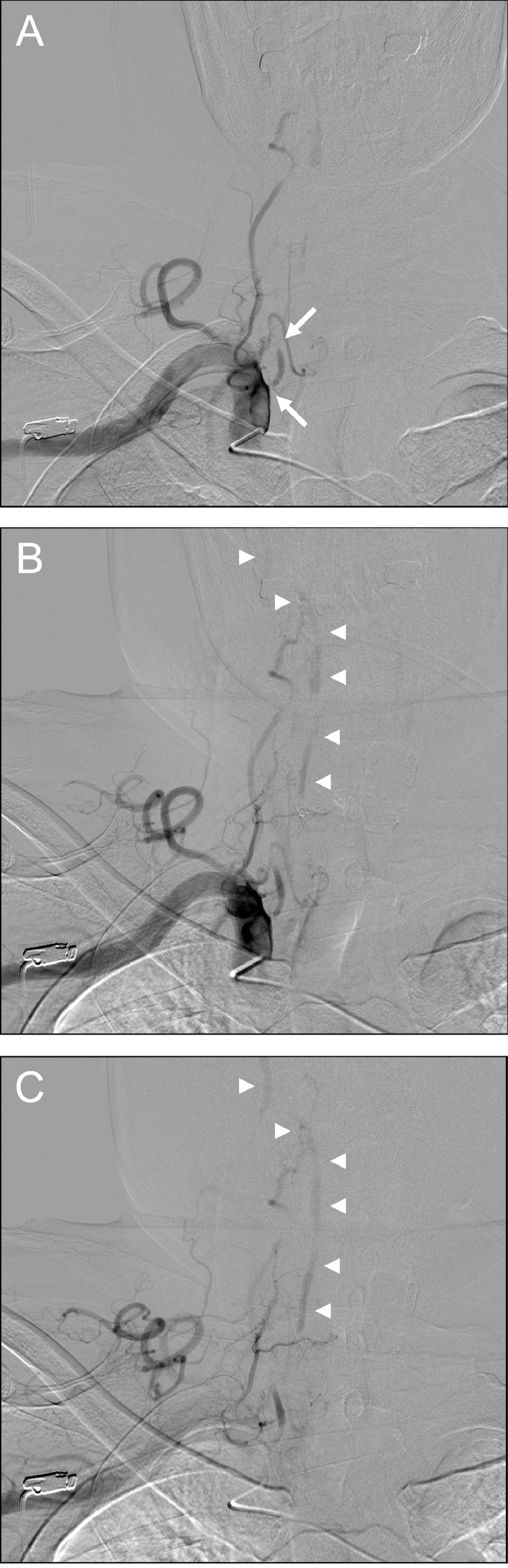
Digital subtraction angiography revealing severe stenosis (functional obstruction) at the origin of the right vertebral artery with stagnant blood flow (arrows) (A). Distal antegrade flow of the right vertebral artery (arrow heads) was supplied via collateral pathways from the deep cervical artery (B, C).

We started the patient on an argatroban (anticoagulant; direct and selective thrombin inhibitor) and edaravone infusion, cilostazol (200 mg/day) and statin, and rehabilitation. Digital subtraction angiography (DSA) revealed severe stenosis (functional obstruction) at the origin of the right VA with stagnant blood flow (Fig. 2A). Peripheral blood flow of the right VA was supplied via collateral pathways from the deep cervical artery, of which some flowed retrogradely toward the origin causing the stagnation of blood flow (Fig. 2B, C). Peripheral blood flow of the right VA was also provided via collateral vessels from the muscular branch of the right occipital artery. There was another stenotic lesion at the origin of the left VA, but no other embolic sources. We diagnosed VASS, and the patient was discharged with no symptoms after 10 days.

Only 18 days after discharge, the patient developed vertigo and was transferred to our hospital by ambulance where MRI demonstrated recurrent right cerebellar infarction. He was readmitted to our hospital and restarted on an intravenous infusion of argatroban and edaravone, with rehabilitation. Antiplatelet therapy was changed from cilostazol to dual antiplatelet therapy with aspirin (100 mg/day) and clopidgrel (75 mg/day). We discussed the treatment plan with the patient and elected to perform angioplasty of the occlusive lesion or embolization of the stump should cerebral infarction recur. Fortunately, he fully recovered and was discharged 11 days after the recurrence. One month after the recurrence, dual antiplatelet therapy was changed to a single antiplatelet agent (clopidgrel). He continued on this antiplatelet therapy for 7 years, at which point a follow-up MRI assessment showed no further recurrence including asymptomatic lesions.

## Discussion

Herein, we report a rare case of VASS treated with antiplatelet therapy (clopidgrel) with a 7-year follow-up period. VASS is characterized by artery-to-artery embolism after an occlusive lesion of VA origin. Embryologically, VA has the potential for numerous anastomoses, and a VA stump may be formed after VA origin occlusion [1]. Moreover, thrombus resulting from the stagnation of blood flow and a low-flow state arising from a VA stump could lead to distal embolism. Previous studies described the presumptive mechanism of VASS as the distal limit of propagated thrombosis, and an embolism of the stagnating clot fragment and a low-flow state caused by collateral flow from deep cervical arteries [1,2].

Kawano et al. proposed the following diagnostic criteria for VASS: (1) acute ischemic stroke in the posterior circulation; (2) VA origin occlusion identified on MRA, duplex ultrasound, computed tomography angiography, and/or DSA; (3) distal antegrade flow in the ipsilateral VA; and (4) the absence of other causes of ischemic stroke [2]. The presence of distal antegrade flow, even though the VA origin is occluded, is a key point in the radiological diagnosis of VASS (Figs. 1B, 2). The VASS prevalence following posterior circulation stroke was previously reported to be 1.4%, with a risk of recurrence in 25% of cases with ischemic stroke in the acute phase, and to be associated with unfavorable outcomes in 25% [2]. There have also been reports of fatal cases such as basilar artery occlusion associated with VASS [3]. Our patient experienced recurrence just 1 month after the initial event and required re-treatment. Although we considered endovascular intervention in the case of any further ischemic events, this has fortunately not yet been necessary.

The optimal treatment for VASS has not been established. Endovascular treatment of VASS has been reported for the prevention of recurrence and as reperfusion therapy for acute ischemic stroke [1,3], but antithrombotic therapy remains the cornerstone of treatment. However, previous case reports have used antiplatelet and anticoagulant therapy [4,5]. Our patient had no recurrence during 7 years with antiplatelet therapy, but it is unclear why this was so effective although it is possible that the mild symptoms experienced on both occasions reflected a small thrombus. Further research is needed to determine the optimal antithrombotic agents in the treatment of VASS.

## Conclusion

Herein, we report a rare case of VASS with a 7-year follow-up period. Improved knowledge about VASS is necessary for the diagnosis and treatment of posterior circulation stroke. Future investigations are needed to establish the optimal antithrombotic therapy for the treatment of VASS.

## Authorship contribution statement

Michiru Katayama: Validation, Writing - review & editing. Hideki Endo: Conceptualization, Methodology, Validation, Formal analysis, Investigation, Resources, Data curation, Writing - original draft, Writing - review & editing, Visualization, Project administration. Megumi Matsuda: Validation, Formal analysis, Investigation, Data curation, Writing - review & editing. Kenji Kamiyama: Supervision. Toshiaki Osato: Supervision. Hirohiko Nakamura: Supervision, Project administration.

## Ethical statement

All procedures performed in studies involving human participants were in accordance with the ethical standards of the institution and/or national research committee and with the 1964 Helsinki declaration and its later amendments or comparable ethical standards. The study was approved by the Ethics Committee of Nakamura Memorial Hospital (No. 2022042201), and informed consent was obtained from the patient.

## Funding

This research did not receive any specific grant from funding agencies in the public, commercial, or not-for-profit sectors.
